# Incidence, severity and distribution of Cassava brown streak disease in northeastern Democratic Republic of Congo

**DOI:** 10.1080/23311932.2020.1789422

**Published:** 2020-07-16

**Authors:** Honoré Muhindo, Sabrine Yasenge, Clérisse Casinga, Médard Songbo, Benoît Dhed’a, Titus Alicai, Justin Pita, Godefroid Monde

**Affiliations:** 1 Laboratory of Plant Virology & West African Virus Epidemiology (WAVE-East DR Congo), Institut Facultaire des Sciences Agronomiques de Yangambi (IFA-Yangambi), Kisangani, BP 1232 Kisangani, DRC; 2 Faculty of Sciences, Department of Plant Biotechnology, University of Kisangani, BP 2012 Kisangani, DRC; 3 International Institute of Tropical Agriculture, IITA-Kalambo, Bukavu, DRC; 4 National Agriculture Research Organization (NARO), National Crops Resources Research Institute (NaCRRI), Uganda; 5 West African Virus Epidemiology (WAVE-Abidjan), Université Félix Houphouët-Boigny (UFHB), BP V34 Abidjan 01, Côte d’Ivoire; 6 Food Engineering and Biotechnology, Middle East Technical University, Ankara, Turkey

**Keywords:** CBSD, DRC, CBSV, UCBSV, Haut Uélé, Ituri

## Abstract

Cassava fields were prospected from two provinces of the Democratic Republic of Congo (Ituri and Haut Uélé) to evaluate the ampleness of Cassava brown streak disease (CBSD) infection. CBSD pressure was determined by assessing the incidence, severity, whitefly abundance and distribution of the disease viruses in the surveyed provinces. A duplex RT-PCR was performed for the simultaneous detection of Ugandan Cassava brown streak virus (UCBSV) and Cassava brown streak virus (CBSV) on 56 cassava leaves sampled in the study area. Our results show a high field CBSD incidence contrasted to a low severity in both provinces. CBSD severity was similar in both provinces (mean disease severity 2). High densities of whitefly were recorded in Ituri province (10 adult whiteflies plant^−1^) than in Haut Uélé where density was 5 adults plant^−1^. However, no relation has been found between whitefly density and CBSD incidence and severity on cassava leaf, root and stems. Molecular analysis showed the incidence of single infections of UCBSV was greater than single infections of CBSV and mixed infections of UCBSV and CBSV. Disease incidence was greater in Ituri than in Haut Uélé; molecular incidence was lower than field incidence. Our results raise the need for appropriate CBSD control strategies in DRC.

## 1. Introduction

**Figure 1.**
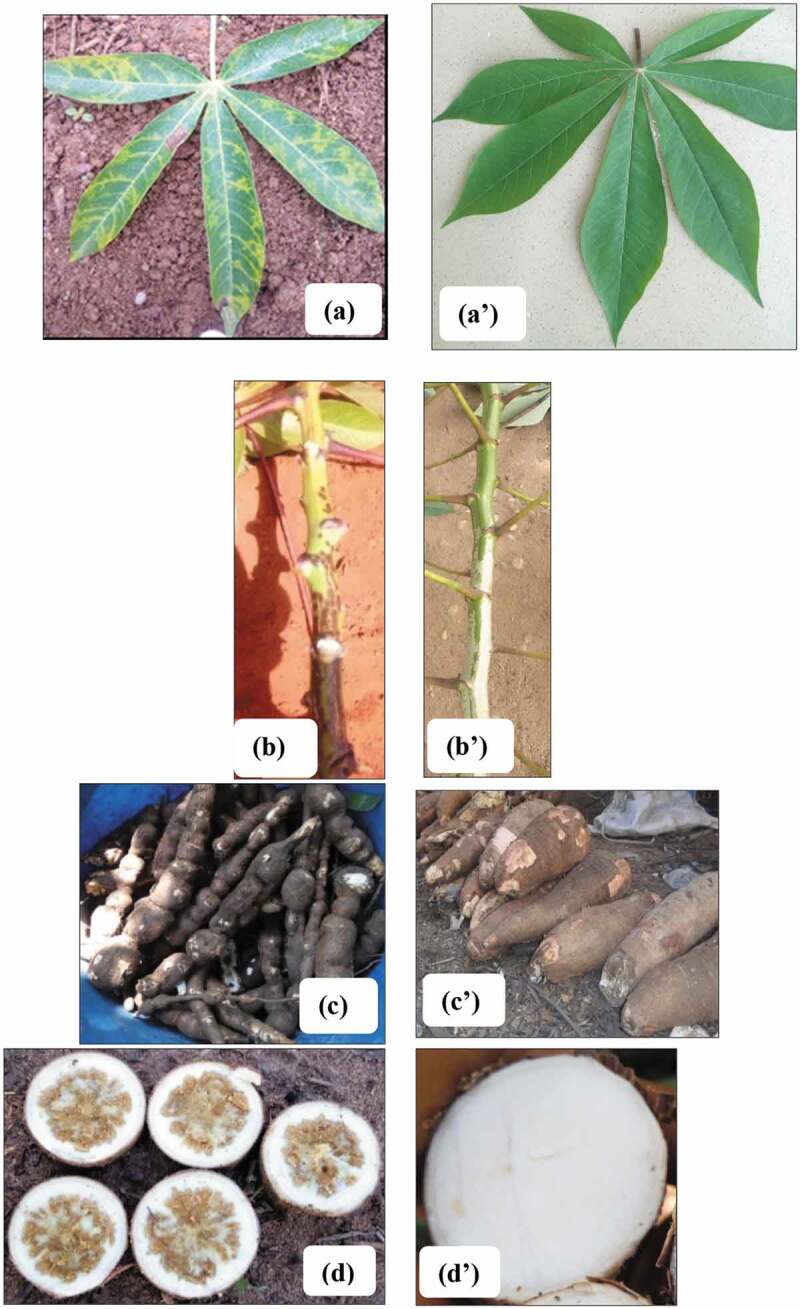
Typical CBSD symptoms observed on cassava leaves (a), stem streaking (b), root constriction (c), and root necrosis (d) and their asymptomatic equivalents (a’), (b’), (c’), (d’).

Cassava brown streak disease (CBSD) has been identified as the most devastating pathogen of cassava in East and Central Africa (Mohammed et al., 2012). CBSD-infected cassava plants are characterized by feathery chlorosis along the leaf veins or circular patches of chlorosis between the veins (Figure 1(a)), brown necrotic streaks on the stem (Figure 1(b)) that results in stem dieback in severe cases, necrosis and occasional radial constrictions of the tuberous roots (Figure 1(c)), necrosis of the pulp (Figure 1(d)), and reductions in starch and cyanide content (Patil et al., 2015).

CBSD is caused by two distinct viruses: *Cassava brown streak virus* (CBSV) and *Ugandan cassava brown streak virus* (UCBSV) that generally cause similar root necrosis in infected plants (Bakelana et al., 2018; Legg et al., 2011; Masinde et al., 2016; Mbanzibwa et al., 2009; Ndunguru et al., 2015; Vanderschuren et al., 2012; Winter et al., 2010). CBSD-causing viruses are semi-persistently transmitted by the whitefly *Bemisia tabaci* (Gennadius) (Njoroge et al., 2017), however, aphids are been identified as potential vectors of CBSV (Ateka et al., 2017; Mulenga et al., 2018).

CBSD symptoms were first reported in the western Democratic Republic of Congo (DRC) by Mahungu et al. (2003) and evidence of CBSD was reported by Mulimbi et al. (2012) and Casinga et al. (2018) using molecular analysis of field samples.

Outbreaks of CBSD in the early 2000s were reported from locations >1000 km inland at moderate altitudes (>1000 m) in several countries around Lake Victoria, including Uganda (Alicai et al., 2007), western Kenya (Masinde et al., 2016), and northern Tanzania (Legg et al., 2011). Over the last 10 years, CBSD has spread to other countries in East and Central Africa, such as Rwanda, Burundi, Congo, DRC, and South Sudan (Alicai et al., 2016; Bigirimana et al., 2011; Mulimbi et al., 2012). In countries where it is already established, CBSD is the main cause of losses in cassava production.

Currently, eastern DRC is at the leading edge of the pandemic spreading westwards from East Africa (Legg et al., 2011); however, the presence of CBSD and CBSVs and the effectiveness of phytosanitary and quarantine measures in large areas of eastern DRC have not yet been assessed. Thus, there is an urgent need to improve understanding of CBSD epidemiology to improve disease awareness alerts and develop strategies for its control in regions where cassava is cultivated. The aim of this study was to quantify incidence, severity, whitefly abundance and the distribution of CBSD in northeastern DRC.

## 2. Materials and methods

### 2.1. Field surveys

Surveys of CBSD in the major cassava growing areas of Haut Uélé and Ituri provinces (Figure 4) in northeastern DRC were conducted in 2016/2017, based on the harmonized protocol described by Sseruwagi et al. (2004). Data were collected using the iForm app and field geolocations were recorded using a GPS (Garmin 62 S).

We prospected 100 cassava fields (plants were 6 to 12 months old), where fields were sampled at intervals of at least 10 km between fields that were accessible by vehicle along roads. In each field, we assessed leaf CBSD symptoms in 30 × 3–10 months-old plants of the dominant cultivated variety along two transects that extended diagonally from opposing corners of the field and intersected at the field-center.

Foliar symptoms (Figure 1(a)) were classified using a severity scale from 1 to 5, where 1: no visible symptoms; 2: mild vein yellowing or chlorotic blotches on some leaves; 3: pronounced/extensive vein yellowing or chlorotic blotches on leaves, but no lesions or streaks on stems; 4: pronounced/extensive vein yellowing or chlorotic blotches on leaves and mild lesions or streaks on stems; and, 5: pronounced/extensive vein yellowing or chlorotic blotches on leaves and severe lesions or streaks on stems, defoliation and dieback (Alicai et al., 2016).

**Figure 2.**
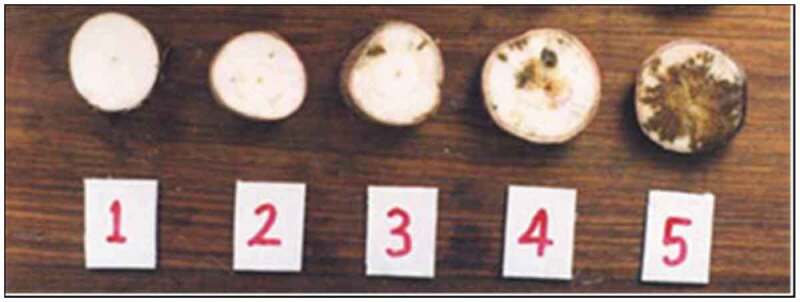
**Scoring system used to assess CBSD root necrosis. 1 = asymptomatic healthy root; 2 = less than 2% of tubers tissue necrosis; 3 = 2–5% tubers tissue necrosis; 4 = 30–40% tubers tissue necrosis; 5 = more than 50% of tubers tissue necrosis (Bakelana** et al.**, 2018**).

Ten plants (>10 months-old) were selected at random along the two transects and we assessed their roots for constriction (Figure 1(c)) and necrotic spots by making cross sections across each tuber (Figure 1(d)). Tuber necrotic spots were scored on a scale of 1 to 5 (Figure 2), where 1: no visible symptoms; 2: <2% necrosis; 3: 2–10% necrosis; 4 = 30–40% necrosis; 5: >50% necrosis (Bakelana et al., 2018). CBSD stem symptoms (Figure 1(b)) were assessed for presence (+) or absence (-) of brown streaks.

We collected 56 leaf samples, such symptomatic and asymptomatic third fully expanded leaf material from plant for virus analysis at NaCRRI-Uganda Laboratory (42 leaves were collected from Ituri and 14 from Haut Uélé). These leaf samples were pressed between pieces of newspaper and placed in a herbarium press, and stored at room temperature prior to molecular analysis.

Disease incidence was calculated as the proportion of sampled plants (percent) that showed disease symptoms, and scores for symptom-free plants were omitted in the calculation of mean incidence and severity for each cultivar and location (Masinde et al., 2016).

### 2.2. CBSD vector abundance

Abundance of the principal vector (adult whitefly, *Bemisia tabaci* Gennadius) on the 5 apical leaves of each of the 30 plants per field that had been assessed for disease symptoms was recorded.

### 2.3. Molecular analysis of CBSD-causing viruses

RNA was extracted from the collected 56 leaf samples using a modified CTAB protocol described by Chang et al. (1993). Resultant RNA pellets (N = 56) were re-suspended in 200 µl of deionised water and stored at −80°C prior to the analysis of CBSD-causing viruses.

Double-stranded cDNA was synthesized from each of the RNA extracts using RevertAid First-Strand Synthesis kit (Thermo Fischer scientific, USA, Biometra-2) according to the manufacturer’s instructions. The extracted cDNA was amplified using specific primers (CBSDDF2 [Fwd 5ʹ-GCTMGAAATGCYGGRTAYACAA-3ʹ] 437 bp UCBSV and CBSDDR [Rev 5ʹ-GGATATGGAGAAAGRKCTCC-3ʹ] 343 bp CBSV), as described by Mbanzibwa et al. (2011), for the simultaneous detection of UCBSV and CBSV in a duplex RT-PCR. The thermocycling conditions were 94°C for 30 s (1 cycle, initial denaturation), then 94°C for 30 s, 51°C for 30 s, 72°C for 30 s (30 cycles, annealing), and 72°C for 10 min (1 cycle, final extension), with a holding step at 4°C. The PCR products were then electrophoresed using a 1.2% agarose gel stained with 5 µl of ethidium bromide and run at 85 V for 1 h in 1x Tris-Acetate-EDTA (TAE) buffer (pH: 8). The gels were visualized under UV light and photographed using a gel documentation system (Syngene U-GENIUS).

## 3. Results

### 3.1. CBSD incidence

Incidence of CBSD in cassava leaf and tuber material was greater in Haut Uélé (55.6 and 26.7%, respectively) than in Ituri (28.0% and 11.3%, respectively) (Table 1). Most cassava cultivars planted in Haut Uélé and Ituri were infected with CBSD.

**Table 1.** Incidence and severity of CBSD, and adult whitefly abundance

Province	Location	Cultivar	Varieties field^−1^	CBSD					Adult whitefly plant^−1^
				LS (1–5)	LI (%)	SS	RS (1–5)	RI (%)	
Haut Uélé	Durba	6 mois	1	2	80	-	1	0	5
	Durba	Bandande	1	1	33.3	-	2	10	0
	Gamo	Goigoi	1	2	33.3	-	1	0	1
	Bevrendi	Omo	1	2	66.7	-	1	0	3
	Kaliva	TMS 419	1	2	70	+	3	40	1
	Isiro, Bingo	FAO	8	2	50	-	2	30	15
	Gobei, Bavemo	Mamawela	5	1	0	-	1	0	2
	Kandawu, Socopa	Modiri	7	1	0	-	1	0	7
		**Mean**	-	**2**	**55.6**		**2**	**26.7**	**5**
Ituri	Sililo	ACODI	1	2	3.3	-	1	0	1
	Araka, Aru	Adulebanda	2	3	13.3	+	2	10	47
	Livu	Ariwara	1	2	20	+	1	0	1
	Nioka (INERA)	Groupe Agricole	1	2	6.7	-	2	10	0
	Vobo	INEAC	1	2	3.3	-	1	0	2
	Gerau	Kakwa	1	3	13.3	+	2	10	0
	Fataki	Lando	1	2	3.3	-	1	0	3
	Nyorinwa	Mayaya	1	2	46.7	+	1	0	1
	Alivio-amagona	Sawasawa	1	2	53.3	+	2	10	29
	Mambasa centre	Pano	1	2	100	+	2	20	3
	Azanovwa	Waiva	1	2	13.3	+	1	0	2
	Suranyama	Rav	1	2	6.7	-	1	0	8
	Bandisango	TMS 419	1	3	6.7	+	2	10	3
	Takumanza	FAO	8	2	10	+	2	10	5
	Chubu, War	Nyagota	5	3	55.9	+	2	10	4
	Ariwara	Olamu	4	3	92.5	+	1	0	31
	Nyapala, Lenga	Nyapamitu	8	1	0	-	1	0	4
		**Mean**	-	**2**	**28.0**		**2**	**11.3**	**10**

LS: Disease severity in leaf material; LI: disease incidence in leaf material; SS: disease severity in stem determined as presence (+) or absence (-) of symptoms; RS: disease severity in root material; RI: disease incidence in root material.

### 3.2. CBSD severity

Mean CBSD severity was similar in Haut Uélé and Ituri (severity index: 2) (Table 1). CBSD symptoms were observed on leaf, stem, and tuberous root material in ‘TMS 419ʹ in Haut Uélé, while in Ituri, CBSD symptoms on leaf, stem, and tuberous root material were recorded from 7 of 17 cultivars. Symptoms in other cultivars were recorded from leaf or root material. In Ituri, 16 of 17 plants infected with CBSD presented symptoms on leaves, 11 exhibited brown streaks on the stem, while eight showed necrosis of the tuber pulp.

In Haut Uélé, five of the eight infected cultivars showed symptoms on leaf material (5/8), one showed brown streaks on the stem (1/8), and three exhibited necrosis in the roots (3/8). Our results show that there were positive associations between root incidence and root severity (r = 0.92, p ≤ 0.01), leaf incidence and leaf severity (r = 0.75, p ≤ 0.01), leaf severity and stem severity (r = 0.71, p ≤ 0.01), CBSD root severity and stem severity (r = 0.57, p ≤ 0.05) (Table 2).

**Table 2.** Correlation coefficients between whitefly abundance and disease incidence (inc) and severity (sev) in leaf and root material

Variable	Leaf.inc	Leaf.sev	Root.inc	Root.sev	Stem.sev	Whitefly.mean
Leaf.inc	1	0.75**	0.38	0.35	0.49	0.45
Leaf.sev	0.75**	1	0.37	0.46	0.71**	0.41
Root.inc	0.38	0.37	1	0.92**	0.45	0.16
Root.sev	0.35	0.46	0.92**	1	0.57*	0.08
Stem.sev	0.49	0.71**	0.45	0.57*	1	0.13
**Whitefly.mean**	**0.45**	**0.41**	**0.16**	**0.08**	**0.13 ^ns^**	1

**p < 0.01; *p < 0.05.

### 3.3. Whitefly abundance

High densities of whitefly were recorded in Ituri province, especially on “Adulebanda”, “Olamu”, “Sawasawa” cultivars that had densities of 47, 31, and 29 adult whiteflies plant^−1^, respectively. In contrast, mean density of whitefly in Haut Uélé was <10 adult whiteflies plant^−1^ (Table 1). Our results show that whitefly density varied among the cultivars. There was no association between whitefly density and CBSD incidence and severity on cassava leaf, root and stems (Table 2).

### 3.4. CBSD-causing viruses

**Figure 3.**
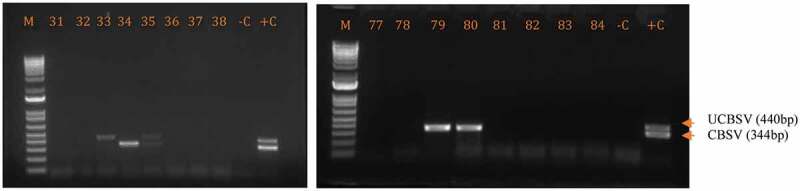
Gel photo showing the amplification of CBSVs in the test samples. M = DNA size marker 1Kb, -C = negative control and +C = positive control and 33–38 and 77–84 are tested samples.

Among the 56 cassava leaf samples tested by RT-PCR, 10 tested positive for CBSD-causing viruses as shown in Figure 3; eight samples were collected from Ituri (19% incidence) and two from Haut Uélé (14.29% incidence). Incidence of CBSD-causing viruses was greatest as single infections of UCBSV (70%) that were more prevalent than single infections of CBSV (20% incidence) in the two provinces; the lowest incidence was for mixed infections of UCBSV + CBSV (10%).

**Figure 4.**
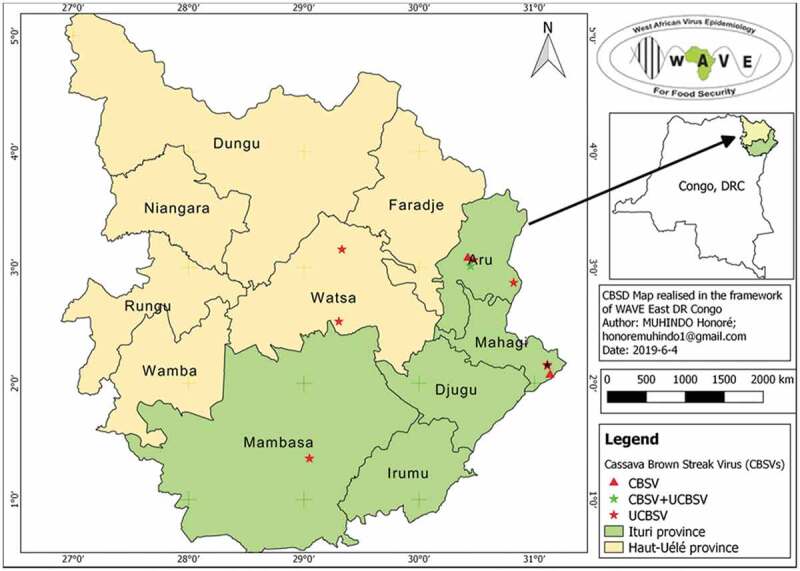
CBSD distribution in Haut Uélé and Ituri provinces, northeastern DRC.

Distribution of UCBSV was more widely distributed in Haut Uélé and Ituri than CBSV and UCBSV + CBSV (Figure 4).

### 3.5. Discussion

The present study shows that the provinces of Haut Uélé and Ituri are moderately infected by CBSD. Our results showed that almost all cassava plants tested, which were distributed across the provinces of Haut Uélé and Ituri, were diseased, with low levels of visible symptoms (severity score 2) on leaves, stems, and tuberous roots. However, most plants tended to show symptoms on either leaves or roots (Table 1). These results suggest that farmers use uncontrolled planting material and that this contributes to extensive disease propagation (Legg et al., 2014).

Similar visible levels of severity have been observed by Bigirimana et al. (2011) in areas of Burundi that border Lake Tanganyika. We found that CBSD symptoms and susceptibility varied among cultivars, supporting similar observations in Kenya (Hillocks & Jennings, 2003; Masinde et al., 2016; Mohammed et al., 2012; Pariyo et al., 2015). Pariyo et al. (2015) classified cassava cultivars according to the level of resistance to CBSD. According to their classification, we found that “FAO” and “Sawasawa” were highly susceptible in Haut Uélé and in Ituri, respectively; it should be noted that these cultivars have been bred for resistance to cassava mosaic disease (CMD).

The positive associations between disease incidence and severity in leaf and root material, between disease severity in leaf and stem material, and between disease severity in the root and stem material indicate that severity of CBSD affects tuber health. Similar case was found in western Kenya, where Masinde et al. (2016) reported a positive association between CBSD and tuber loss. The associations between incidence and severity of CBSD infection highlight the need to improve CBSD resistance in cassava varieties to increase productivity in DRC.

We found no correlation between whitefly density and CBSD incidence. This may be due to the lower mean adult whitefly density (10 whiteflies plant^−1^ recorded in Ituri) compared in other CBSD outbreak areas, such as the African Great Lakes region, where Bigirimana et al. (2011) found up to 38 whiteflies plant^−1^. In our case, it is likely that the super abundance of whitefly perpetuated by climate conditions in northeastern DRC may negatively affect cassava when inoculum pressure is high. This means that CBSD in Ituri and in Haut Uélé provinces is propagated mainly through the use of infected cuttings for planting. Also, it may be due to the low inoculum pressure (titer of virus in the plant) in the cultivation zones (Maruthi et al. 2005).

RT-PCR analyses showed that UCBSV was more prevalent than CBSV in Haut Uélé and in Ituri provinces, where incidence was greater in Ituri (19%) than in Haut Uélé (14.29%) (Table 3). These levels of incidence derived from the molecular analyses were lower than those recorded from field observations. The high number of samples that tested negative for the two CBSD-causing viruses may be a result of infection with a different pathogen that produces CBSD-like symptoms. Perhaps primers used are not very specific or are not focused on the conserved region of the virus, as RNA-viruses like CBSV/UCBSV tend to mutate quickly. A comprehensive sequence analysis is needed to understand the reason for this high proportion of negatives samples. These results suggest the screening using additional specific primers.

**Table 3.** Detection of virus species in cassava samples from Ituri (N = 42) and Haut Uélé (N = 14), northeastern DRC

Province	Territory	Location	Latitude	Longitude	Altitude (m asl)	Cultivar	Virus
Ituri	Aru	Ndere barrière	3.06510 N	30.47477E	1267	Olamu	UCBSV
	Aru	Ariwara	3.08167 N	30.42276E	1257	Olamu	CBSV
	Aru	AlivioAmagona	3.01227 N	30.44464E	1277	Sawasawa	CBSV+UCBSV
	Aru	Aru-Centre	2.86853 N	30.8197E	1345	Adulebanda	UCBSV
	Mahagi	War	2.16207 N	31.11142E	1004	Nyagota	UCBSV
	Mahagi	Mahagi-Port	2.07203 N	31.13533E	640	Nyagota	CBSV
	Mahagi	Chubu	2.15043 N	31.1115E	1044	Nyagota	UCBSV
	Mambasa	Mambasa Centre	1.35558 N	29.0486E	895	Pano	UCBSV
Haut Uélé	Watsa	Bevrendi	2.53374 N	29.30353E	1114	Omo	UCBSV
	Watsa	Kaliva	3.15556 N	29.33004E	839	TMS 419	UCBSV

In general, we found a wide distribution of CBSD across cassava growing areas in northeastern DRC, highlighting the possibility that other low-altitude areas in central Africa, which were previously thought to be unaffected by CBSD, may see increases in prevalence in CBSD. Mohammed et al. (2012) associated the incidence of CBSD in mid-altitude areas with the coastal endemic virus CBSV and the highland isolate UCBSV. In our study, two isolates of UCBSV were found to be prevalent at low altitude (Haut Uélé) and high altitude (Ituri), and more prevalent than CBSV, confirming results reported by Mulimbi et al. (2012) in Nord Kivu province, and by Casinga et al. (2018) in Ituri province. Similarly, in Uganda, which neighbors DRC to the northeast, UCBSV isolates are more common than CBSV (Mbanzibwa et al., 2009).

The high levels of CBSD incidence recorded from fields in Haut Uélé (55.6%) and in Ituri (28%) may be attributed to the planting of infected cuttings sourced from the farming community (Legg et al., 2014). Given this disease damages root pulp moreover decreases the consumer and market value of cassava (Mulenga et al., 2018), we suggest that CBSD management strategies are implemented with urgency in the two provinces, including controlling the distribute, use and propagation of infected planting material (Legg et al., 2014).

Our study supports the hypothesis that CBSD continues to spread from East to West Africa through countries in central Africa. Confirmation of CBSD in eastern DRC (Casinga et al., 2018; Mulimbi et al., 2012) highlights the necessity for a more comprehensive epidemiological study of CBSD and an intensive investigation of this disease in the region. Analysis of factors, such as virus diversity, whitefly population dynamics, cropping system, and source of planting materials, is recommended to increase understanding of the epidemiology of CBSD in DRC urgently required to design and implement effective strategies to control the spread, incidence, and severity of CBSD in West Africa.

## Figures and Tables

**Figure:**
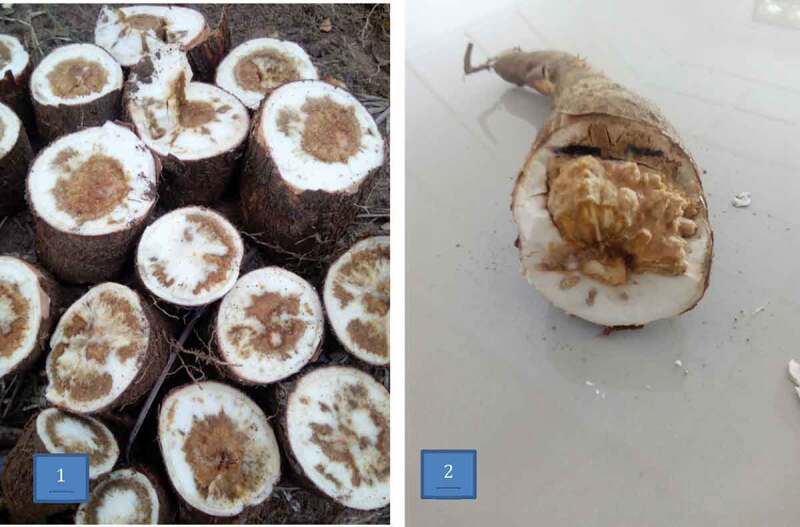


## Cover Image

Source: Author.
